# Gender difference in the authorship of top anesthesiology and pain journals: a protocol for retrospective bibliometric study

**DOI:** 10.3389/fmed.2026.1945719

**Published:** 2026-08-26

**Authors:** Bo Jiao, Liyun Deng, Mingyuan Chen

**Affiliations:** 1 Department of Anesthesiology, State Key Laboratory of Oral Diseases, National Clinical Research Center for Oral Diseases, West China Hospital of Stomatology, Sichuan University, Chengdu, China; 2 Department of Anesthesiology, West China Hospital, Sichuan University, Chengdu, Sichuan, China; 3 Department of Pathology, School of Medicine, The Affiliated Hospital, UESTC Chengdu Women’s & Children’s Central Hospital, Chengdu, Sichuan, China

**Keywords:** anesthesiology and pain, authorship, bibliometric study, gender difference, protocol

## Abstract

**Introduction:**

The number of women in medicine has increased, and female scientists play an important role in medical research. However, bias in research publication and funding persists, which may influence the representation of women in medical studies. This study aims to explore women's representation in the authorship of top-five high-impact anesthesiology and pain journals over the past ten years, particularly during the COVID-19 pandemic period.

**Methods:**

This retrospective bibliometric study will be performed according to relevant reporting recommendations for observational studies. The five most influential anesthesia and pain management journals will be selected based on their impact factor and most recent H-index (2024 Journal Citation Reports). Two researchers will search the Web of Science (WOS) Core Collection database for publications in these journals between 1 January 2015 and 31 December 2024 based on the journal-title. Microsoft Excel 2016 and SPSS (version 26.0, IBM) will be used for data storage and analysis. Statistical analysis will be performed according to various purposes, and a regression model will be applied to investigate the correlation between different variables.

**Discussion:**

The academic status of female authors in the fields of anesthesiology and pain medicine will be comprehensively summarized from multiple perspectives through this research. This study will identify the top-five anesthesiology and pain journals and analyze authorship gender disparities, providing key insights into women's contributions to the field and guiding future research.

## Introduction

Recent studies have indicated that the representation of women in medicine has increased, and the Association of American Medical Colleges (AAMC) reported that women have become an increasingly significant component of the physician workforce. However, gender representation varies substantially across specialties, and anesthesiology has the notable gender imbalance (1). Representation of women in medical journals has always been a topic of interest, as authorship is important for promotion, professional prestige, and future academic direction. Despite this, women remain underrepresented in academic practice. There were long-standing biases in funding bodies and research organizations, particularly in high-impact journals (2–4).

In specialties such as critical care (5), cardiology (6), rheumatology (7), Oncology (8), and surgery (9), gender disparities in female authorship have been reported, especially in high-impact journals. Miller et al. had found that the proportion of female authors in Anesthesiology and Anesthesia& Analgesia journals increased from 20.5% to 30.2% (10). However, whether these previously observed increases in female authorship are generalizable across a broader range of high-impact anesthesiology and pain medicine journals remains unclear. In addition, it remains unknown whether gender representation varies based on authorship position, research characteristics, and geographic factors, and whether major influence such as the COVID-19 pandemic were associated with changes in authorship gender. For the advancement of women in anesthesiology and pain medicine, it is crucial to recognizing gender differences in authorship. This project provides a comprehensive retrospective bibliometric analysis of the gender disparities in authorship of influential anesthesiology and pain journals.

This study aims to examine the representation of women in authorship of top-five high-impact anesthesiology and pain journals over the past ten years, included the COVID-19 period. The study will investigate the proportions of first author gender among all first authors and the proportions of last author gender among all last authors.

## Methods and analysis

### Study design

This study was designed as a retrospective bibliometric analysis in which data are sourced from the published literatures. In this case, the data will be extracted from the top-five high-impact anesthesiology and pain journals over the course of ten years (1 January 2015 to 31 December 2024). Furthermore, the data will be collected from publications that can be searched in the database. Therefore, ethical consent is not required. This retrospective bibliometric study will be conducted in accordance with the Strengthening the Reporting of Observational Studies in Epidemiology (STROBE) reporting guideline (11).

### Study selection

The included journals will be selected according to predefined criteria. The journals primarily focusing on anesthesiology and pain medicine will be identified from the 2024 Journal Citation Reports (JCR). The journals will be ranked according to their 2024 Journal Impact Factor, and the top-five journals with the highest impact factors will be included for analysis. The H-index (https://www.scimagojr.com/journalrank.php) will be additionally reviewed as a supplementary indicator of journal influence. The relevant databases will be accessed on September 10, 2026.

Two researchers will independently search the Web of Science (WOS) Core Collection database, and the search strategy will use the “journal name” field combined with predefined publication years (1 January 2015 to 31 December 2024). The final search will be conducted on September 15, 2026. This study will incorporate publications containing original research and reviews based on the Web of Science Core Collection classification. Editorials, letters, corrections, meeting abstracts, conference report, and other non-research publication types will be excluded. Studies in which the gender of the first author and last author is not distinguished will be excluded. Any studies authored by the working group will also be excluded. Discrepancies in study selection will be resolved through discussion with a senior researcher.

### Outcomes

The primary outcome is the proportions of first author's gender among all first authors. And the secondary outcomes are the proportions of the gender proportions of all last authors. Other secondary outcomes include 1) the association between the proportions of the first author's gender and the proportions of the last author's gender, as well as the region of origin, research topic, study type. 2) the proportions of the first and last author in various degrees. 3) the gender ratio across various years.

### Data collection

Two researchers will independently collect data. Any discrepancies in data collection will be resolved through discussion with a senior researcher. A standardized data extraction form will be applied to collect the data of interest, including journal title, time of publication, the gender of the first and last author (male and female), the degree of the first and last author (e.g., MD, MBSS), the geographic region according to the institutional affiliation of the first and last author (Asia, Europe, North America, South America, Africa, Oceania), the total number of authors in each included study, research topics based on the primary focus of each article (General anesthesia, Cardiothoracic anesthesia, Neurosurgical anesthesia, Orthopedic anesthesia, pediatric anesthesia, Obstetrics and Gynecology Anesthesia, critical care, pain management, basic science), and study type based on the methodology described in the original article (randomized controlled trials, Cohort study, case–control study, Cross-sectional study, and review). The number of publications excluded due to undetermined gender will also be collected.

The gender of first and last authors will be independently investigated by two researchers using publicly available information. First, the gender of the authors will be determined based on their first names and any accompanying biographical information. If the gender cannot be determined by the first name, social media and academic websites, such as X (formerly Twitter) and ResearchGate, will be used to distinguish the gender. The article will be excluded from the study if the gender is unclear or not stated using the method described above. The website (American Association of Medical Colleges) will be accessed to acquire data on physician specialties. And inter-rater agreement for gender classification will be assessed with Cohen's kappa coefficient. Any discrepancies between investigators were resolved by a third senior researcher, who independently evaluate the relevant data.

### Statistical analysis

Microsoft Excel 2016 and SPSS (version 26.0, IBM) will be used for data storage and analysis. Continuous variables will be described with means with standard deviations or medians with interquartile ranges, and categorical variables will be presented as frequencies and percentages. The primary outcome is the gender of the first author. Secondary analysis will assess the gender of the last author. Differences in gender distribution across journals, publication years, geographic regions, research topics, study types, and academic degrees will be assessed using chi-square tests or Fisher's exact tests when appropriate.

Logistic regression analysis will be performed to identify factors associated with female first authorship and female last authorship. For the analysis of female first authorship, independent variables will include last author gender, publication year, geographic region, author degree, research topic, and study type. For the analysis of female last authorship, independent variables will include first author gender, publication year, geographic region, author degree, research topic, and study type. Results will be reported as odds ratios (ORs) with 95% confidence intervals (CIs). And the number of candidate predictors included in each regression model will be determined according to the number of available outcome events. Based on the historical publication number of the selected journals, we anticipate that the 10-year study period will include at least approximately 3000 publications. Therefore, the sample size is sufficient to conduct the planned statistical analysis. Especially, publication year will be considered as a continuous variable in regression models to assess temporal trends in female authorship. In addition, the annual proportions of female first and last authors will be calculated descriptively. The potential effect of the COVID-19 pandemic on gender representation will also be explored. Publications will be categorized into two periods: before COVID-19 (2015–2019) and COVID-19 pandemic period (2020–2024). Differences in female first and last authorship between these periods will be assessed by chi-square tests. In multivariable logistic regression analysis models, COVID-19 period will be included as a binary covariate. Missing data for variables other than author gender will be handled using complete-case analysis. Articles with unclear first or last author gender after assessment using predefined criteria will be excluded from the final analysis.

The significance level for all statistical tests will be set to *P* < 0.05, meaning that results with a probability of occurring by chance of less than 5% will be considered statistically significant.

### Ethics and dissemination

Research ethics approval will not be necessary, given the use of publicly available data and the absence of human subjects. The study will be conducted following the Declaration of Helsinki. The results of this study will be disseminated through scientific conference presentations and research publications. Overall, this study's findings will identify gender disparities and contribute to the knowledge of why such gaps exist as the first step toward informing and addressing issues in diversity and inclusion.

## Discussion

Women are underrepresented in academic medical practice, particularly in men-dominated specialties such as anesthesiology and pain, and plastic surgery (12, 13). This protocol describes a systematic bibliometric approach to evaluate gender representation in authorship within the top-five high-impact anesthesiology and pain medicine journals. By exploring the first and last authorship positions over a decade, this study aims to provide insights into both early-career academic participation and academic leadership representation.

This protocol was developed to address main gaps in the existing literature on gender representation in academic publications. A previous study, performed by Miller et al. (10), mainly focused on gender trends in selected anesthesiology journals. However, this study will investigate multiple high-impact anesthesiology and pain medicine journals and assess gender representation according to authorship position, publication characteristics, and changes over time. Upon successful completion, this research will provide robust evidence regarding the current status and temporal evolution of gender representation in anesthesiology and pain medicine publications. This information may help academic societies, journal editors, and institutions develop targeted strategies to promote equitable opportunities and enhance gender diversity in academic medicine.

There are limitations to the protocol that must be acknowledged. First, there may be some limitations in determining gender based on publicly available data, such as names and gender pronouns. Although this method has been widely used in bibliometric studies, it may introduce misclassification bias, particularly for authors with gender-neutral names, culturally diverse naming systems, or insufficient publicly available information. Therefore, the reported gender distribution should be interpreted cautiously. Second, the journal selection may cause selection bias because this study focuses only on the five highest-impact anesthesiology and pain journals. Therefore, the findings may not be applicable to other anesthesiology and pain journals, particularly lower-impact or regional journals. Third, the scope of this study is limited to top-five high-impact anesthesiology and pain journals, which may limit the generalizability of the study's findings.
